# Simultaneous Determination of 11 Compounds in Gualou Guizhi Granule and Pharmacokinetics Study by UPLC-MS/MS

**DOI:** 10.1155/2017/8451383

**Published:** 2017-07-20

**Authors:** Chengtao Sun, Wen Xu, Yuqin Zhang, Lishuang Yu, Miao Ye, Kedan Chu, Wei Xu, Yu Lin

**Affiliations:** ^1^College of Pharmacy, Fujian University of Traditional Chinese Medicine, Fuzhou, Fujian 350122, China; ^2^Centre of Biomedical Research & Development, Fujian University of Traditional Chinese Medicine, Fuzhou, Fujian 350122, China

## Abstract

A rapid and sensitive ultrafast performance liquid chromatography-tandem mass spectrometry method (UPLC-MS/MS) was developed for the simultaneous determination of 11 compounds in Gualou Guizhi Granule (GLGZG), including liquiritin, isoliquiritin, liquirtin apioside, isoliquiritin apioside, liquiritigenin, isoliquiritigenin, glycyrrhizic acid, glycyrrhetinic acid, paeoniflorin, albiflorin, and paeoniflorin sulfonate in rat plasma. UPLC-MS/MS assay with negative ion mode was performed on a Waters CORTECS C18 (2.1 × 100 mm, 1.6 *μ*m) with the mobile phase consisting of 0.1% aqueous formic acid (A) and acetonitrile (B) in gradient elution at a flow rate of 0.25 mL·min^−1^. The method was linear for all analytes within the detection range (*r* ≥ 0.9597). The inter- and intraday precision (RSD) were 2.21–6.41% and 1.67–6.18%; the inter- and intraday accuracy (recover) were 92.48–114.03% and 90.23–112.04%. And the recovery rate ranged from 81.30% to 108.22%. The matrix effect values obtained for analytes ranged from 88.91% to 113.32%. This validated method was successfully applied to a pharmacokinetics study in rats after oral administration of GLGZG.

## 1. Introduction

Prescription of Gualou Guizhi Granule (GLGZG) was first recorded in “Essentials from the Golden Cabinet” (around 210 AD) [1], which consisted of six herbs, including* Trichosanthes kirilowii *Maxim.,* Paeonia lactiflora *Pall.,* Cinnamomum cassia *Presl.,* Glycyrrhiza uralensis *Fisch.,* Zingiber officinale *Rosc., and* Ziziphus jujuba *Mill. according to yin-yang and wuhsing (five elements) theory of Traditional Chinese Medicine (TCM) in a weight ratio of 10 : 3 : 3 : 3 : 2 : 3.

GLGZG has long been applied clinically to treat muscular spasticity following stroke, epilepsy, or spinal cord injury [2–4]. At present, the granule of GLGZG (Min drug system approval No. S20130001) has been approved to be a standard hospital prescription at Fujian University of TCM Affiliated Second People's Hospital (Fuzhou, China). In recent years, much attention has been paid to its phytochemical and biological studies. Phytochemical studies showed that 104 compounds in GLGZG were identified or tentatively characterized and several bioactive components, such as citrulline, luteolin, puerarin, liquiritin, taxifolin, naringin, formononetin, isoliquiritigenin, 6-gingerol, curcumin, caffeic acid, ferulic acid, jujuboside A, protocatechuic acid, cinnamic acid, catechin, and paeoniflorin, were quantified [5–7]. And pharmacological studies also showed that GLGZG had significant effects of anti-inflammatory [8–10], antioxidation [11], antiexcitotoxicity [12, 13], and antiapoptosis [14, 15] in vitro and in vivo. Meanwhile, to our knowledge, some literature about* Trichosanthes kirilowii *Maxim. [16, 17], protocatechuic acid [18], catechinic acid [19], curcumin [20], 6-gingerol [21], paeoniflorin [22], and isoliquiritigenin [23] proved that they had effect on cerebral ischemia-reperfusion injury.

However, pharmacokinetics (PK) of GLGZG has not been reported. Modern serum pharmacology researches think that figuring out the bioactive components of TCM and its chemical structural formula of metabolic product in human blood is extremely important [24]. Therefore, to support the comprehensive PK of GLGZG, it is important to develop a rapid and sensitive method to simultaneously determine the bioactive components in plasma samples. Our team's previous study showed that there were 42 ingredients including liquiritin, isoliquiritin, liquirtin apioside, isoliquiritin apioside, liquiritigenin, isoliquiritigenin, glycyrrhizic acid, glycyrrhetinic acid, paeoniflorin, albiflorin, and paeoniflorin sulfonate in rat plasma. Thus, above 11 ingredients were selected to assess the PK characteristics of GLGZG in this study [5, 6].

## 2. Materials and Methods

### 2.1. Materials

**Figure 1:**
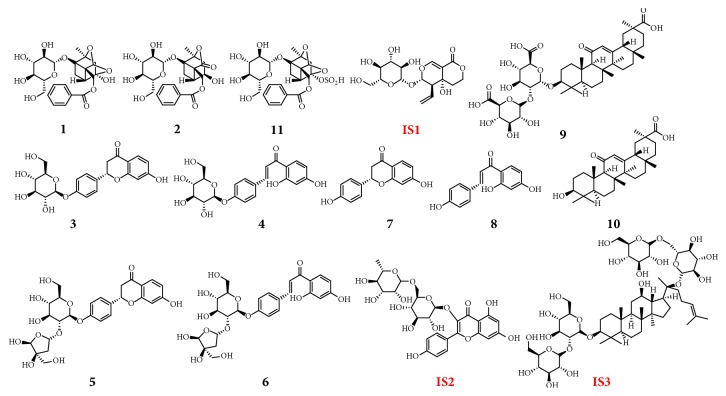
Chemical structures of the eleven standard reference compounds and ISs. 1: paeoniflorin; 2: albiflorin; 3: liquiritin; 4: isoliquiritin; 5: liquiritin apioside; 6: isoliquiritin apioside; 8: liquiritigenin; 9: isoliquiritigenin; 10: glycyrrhizic acid; 11: glycyrrhetinic acid; 12: paeoniflorin sulfonate; IS1: ginsenoside Rb1; IS2: swertiamarin; IS3: kaempferol-3-O-rutinoside.

GLGZG were provided by Fujian University of TCM Affiliated Second People's Hospital (Fuzhou, China). Its voucher specimens were deposited in the College of Pharmacy, Fujian University of TCM. Standard substances (glycyrrhizic acid, glycyrrhetinic acid, isoliquiritigenin, and albiflorin) were purchased from Chengdu MUST Biological Technology Co., Ltd. (Sichuang, China), and standard substances (liquiritin, liquiritin apioside, and isoliquiritin apioside) and internal standards (swertiamarin, kaempferol-3-O-rutinoside, and ginsenoside Rb1) were bought from the Shanghai Tauto Biotech Co., Ltd. (Shanghai, China). Paeoniflorin sulfonate was separated and purified by self, it is confirmed by MS and NMR and its purity was above 98% by HPLC-DAD. Their structures were shown in Figure 1. Methanol and acetonitrile were of HPLC grade and purchased from Merck Co. (Darmstadt, Germany). All other chemical reagents evolved in this research were of analysis grade or better.

All specific pathogen-free (SPF), male Sprague-Dawley (SD) rats, weighing 250 ± 20 g, were bought from Beijing Weitonglihua Experimental Animal Technical Co., Ltd. (Beijing, China), animal qualified number SCXK (Jing) 2012-0001. The principles of laboratory animal care were followed and the study was approved by the Ethics Committee of Fujian University of TCM, China.

### 2.2. HPLC/MS/MS Conditions

Chromatographic separation was performed on an Waters CORTECS C18 (2.1 × 100 mm, 1.6 *μ*m) with the column temperature at 45°C. The mobile phase was composed of 0.1% formic acid in water (A) and acetonitrile (B) with a gradient program (0–0.5 min from 8% to 10% B; 0.5–2.5 min from 10% to 15% B; 2.5–4.0 min from 15% to 60% B; 4.0–5.0 min from 60% to 95% B; 5.0–6.5 min 95% B). The flow rate was 0.25 mL/min and the injection volume was 1 *μ*L.

Mass spectrometry was operated using a Waters (Milford, MA) Xevo TQMS with an electrospray ion source (ESI) in the negative ion multiple reaction monitoring (MRM) mode. The other detailed MS parameters were as follows: drying gas: N_2_ (purity of 99.9%), 15 L/min, nebulizing gas: 50 L/min, capillary voltage: 2.5 kv. The optimized MRM parameters are summarized in Table 1.

### 2.3. Preparation of Standards, Internal Standard, and Quality Control (QC) Samples

Eleven standard stock solutions were prepared individually at concentrations 1 mg/mL by dissolving the substance in methanol. Then their working solutions were serially diluted with methanol with different concentrations used for plotting standard curves. Internal standards stock solution were also prepared in a concentration of 5 *μ*g·mL^−1^ for swertiamarin, 2.5 *μ*g·mL^−1^ for kaempferol-3-O-rutinoside, and 5 *μ*g·mL^−1^ for ginsenoside Rb1 in methanol. All the solutions were stored at 4°C until use.

The calibration curves and quality control (QC) samples were prepared by spiking the appropriate amount of working standards to blank rat plasma. The calibration curves were in the concentration of 0.04–8 *μ*g·mL^−1^ for paeoniflorin, 0.04–10 *μ*g·mL^−1^ for albiflorin, 0.005–1 *μ*g·mL^−1^ for liquiritin, 0.005–0.5 *μ*g·mL^−1^ for isoliquiritin, 0.005–0.5 *μ*g·mL^−1^ for liquirtin apioside, 0.005–0.5 *μ*g·mL^−1^ for isoliquiritin apioside, 0.001–0.2 *μ*g·mL^−1^ for liquiritigenin, 0.001–0.2 *μ*g·mL^−1^ for isoliquiritigenin, 0.05–10 *μ*g·mL^−1^ for glycyrrhizic acid, 0.01–4 *μ*g·mL^−1^ for glycyrrhetinic acid, and 0.05–11.25 *μ*g·mL^−1^ for paeoniflorin sulfonate. QC samples were paeoniflorin (0.1, 2, 8 *μ*g·mL^−1^), albiflorin (0.1, 2, 8 *μ*g·mL^−1^), liquiritin (0.01, 0.2, 0.5 *μ*g·mL^−1^), isoliquiritin (0.01, 0.1, 0.25 *μ*g·mL^−1^), liquirtin apioside (0.01, 0.1, 0.25 *μ*g·mL^−1^), isoliquiritin apioside (0.01, 0.1, 0.25 *μ*g·mL^−1^), liquiritigenin (0.005, 0.1, 0.2 *μ*g·mL^−1^), isoliquiritigenin (0.005, 0.02, 0.05 *μ*g·mL^−1^), glycyrrhizic acid (0.1, 2, 8 *μ*g·mL^−1^), glycyrrhetinic acid (0.01, 1, 2 *μ*g·mL^−1^), and paeoniflorin sulfonate (0.1, 2, 8 *μ*g·mL^−1^).

### 2.4. Sample Preparation

Plasma samples (100 *μ*L of plasma) and IS working solutions (10 *μ*L of swertiamarin, kaempferol-3-O-rutinoside, and ginsenoside Rb1) were combined and briefly vortex-mixed for 3 min followed by the addition of 4-fold acetonitrile. Then the mixture was centrifuged at 12000 rpm for 10 min. The supernatant was transferred to a clean tube and concentrated up to dryness; the residue was dissolved in 100 *μ*L 50% methanol. Finally, 1 *μ*L aliquot was injected into UPLC-MS/MS system for analysis.

### 2.5. Method Validation

#### 2.5.1. Specificity

Specificity of the method was assessed by comparing chromatograms of six different sources of blank rat plasma with the corresponding spiked rat plasma. It was analyzed to investigate the presence of potential interferences from endogenous matrix components.

#### 2.5.2. Linearity, Lower Limit of Quantification (LLOQ), and Lower Limit of Detection (LLOD)

Linearity was evaluated on eight nonzero concentrations and was assessed by weighted (1/*x*^2^) least-squares analysis. The LLOQ and LLOD were defined by signal-to-noise ratio method. LLOQ should be ten times the noise level (S/N ≥ 10) and LLOD should be three times the noise level (S/N ≥ 3). For each target constituent, the LLODs and LLOQs were determined by serial dilution of standard solution under the described UPLC-MS/MS conditions.

#### 2.5.3. Precision and Accuracy

Intra- and interday variations were employed to evaluate the precision of the method. Intraday precision was evaluated in six replicates at three QC levels at one day and interday precision was evaluated at three QC levels at six days. The precision (RSD, %) should not exceed 15% and the accuracy values should be within 15% of the actual values for QC samples.

#### 2.5.4. Recovery and Matrix Effect

The recoveries of the analytes from plasma samples were determined by comparing the peak areas of the analytes in plasma samples after extraction to those of the same concentration of the analytes spiked into the solution extracted from blank plasma samples. The matrix effects were measured by comparing the peak areas obtained from samples with the analytes spiked after extraction, at three QC concentration levels (low, middle, and high), to those obtained from standard solutions at the same concentrations.

#### 2.5.5. Stability

Stability of the analytes from plasma samples was performed by determining three different concentrations (low, middle, and high samples) in six replicates under different conditions. It included short-term stability which was assessed by keeping samples at room temperature (25°C) for 24 h, stability which was measured using samples stored at 4°C for 24 h, and freeze-thaw stability that was determined after three freeze-thaw cycles at −80°C.

### 2.6. Applications in Pharmacokinetics Studies

Six male SD rats, weighing 250 ± 20 g, were provided by Experimental Animal Research Center of Fujian TCM University (Fuzhou, China). And they were housed at controlled environmental conditions (temperature: 23 ± 2°C, relative humidity: 55 ± 10%) with free access to standard laboratory food and water. The rats were fasted for 12 h but with access to water before being given GLGZG (3.6 g·(kg·day)^−1^). Blood samples of 0.3 mL were collected in heparin containing tubes from epicanthic veins of rats by capillary tube before dose (0 h) and at 5 min, 10 min, 20 min, 0.5 h, 1 h, 1.5 h, 2 h, 3 h, 6 h, 9 h, and 12 h. Then they were centrifuged at 12000 rpm for 10 min. The supernatant liquor was collected and frozen at −20°C until use.

## 3. Results and Discussion

### 3.1. Method Validation

#### 3.1.1. Specificity

**Figure 2:**
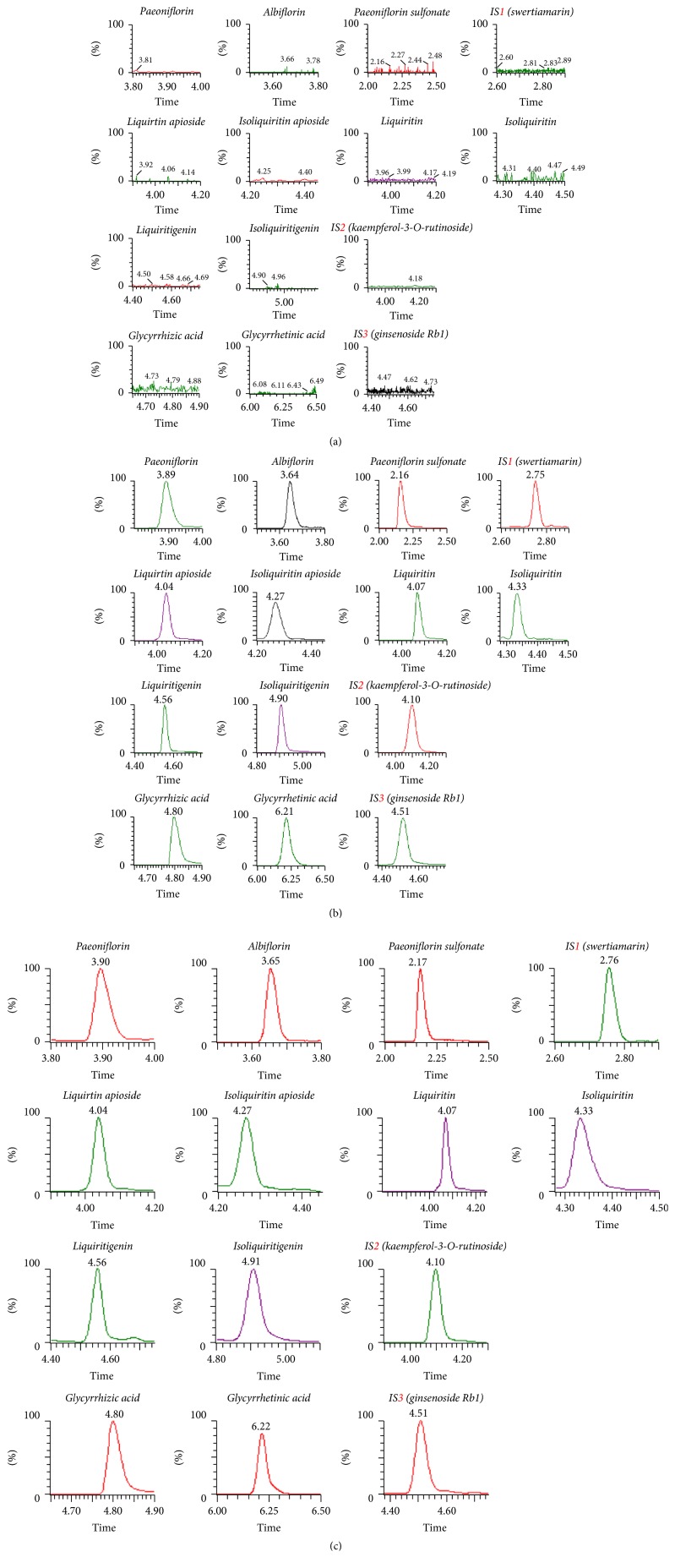
Typical MRM chromatograms GLGZG in rat plasma samples. (a) Blank rat plasma. (b) Plasma spiked with standards mix and IS. (c) Plasma sample after administration of GLGZG at 0.5 h.

Figure 2 was the typical chromatograms for blank plasma, blank plasma spiked 11 standard solutions, and IS working solutions. No obvious interferences from endogenous compound were found at the retention times and at the selected mass transitions of all the analytes and ISs.

#### 3.1.2. Linearity, LLOQ, and LLOD

The regression equations, linear ranges, and correlation coefficients (*r*) of eleven analytes were shown in Table 2. The calibration curves of eleven analytes exhibited good linearity. LLOQ and LLOD of eleven analytes were shown in Table 3.

#### 3.1.3. Precision and Accuracy

Results of the intra- and interday precision and accuracy were listed in Table 4. The intra- and interday precisions were 2.21–6.41% and 1.67–6.18%. The intra- and interday accuracy were 92.48–114.03% and 90.23–112.04%. It was found that, for all the analytes, both the accuracy and precision met the acceptable criterion.

#### 3.1.4. Stability

Results of the stability tests were listed in Table 5. It was shown that all analytes were stable in plasma samples at room temperature (25°C) for 24 h, at 4°C in autosampler for 24 h, and at −80°C within three freeze-thaw cycles.

#### 3.1.5. Recovery and Matrix Effect

Results of the extraction recoveries and matrix effects were summarized in Tables 6 and 7. The extraction recoveries of three concentration levels (low, middle, and high) QCs were 81.30–106.28%, 85.14–108.22%, and 88.65–104.21%. It was indicated that the extraction recovery of this method was efficient, consistent, and reproducible. The matrix effect values obtained for all analytes at three concentration levels (low, middle, and high) QCs were 88.91–113.32%, 91.78–107.86%, and 96.87–110.07%. There was no measurable matrix effect on the ionization of all analytes and ISs and this indicated that the method was acceptable.

### 3.2. Pharmacokinetic Study

**Figure 3:**
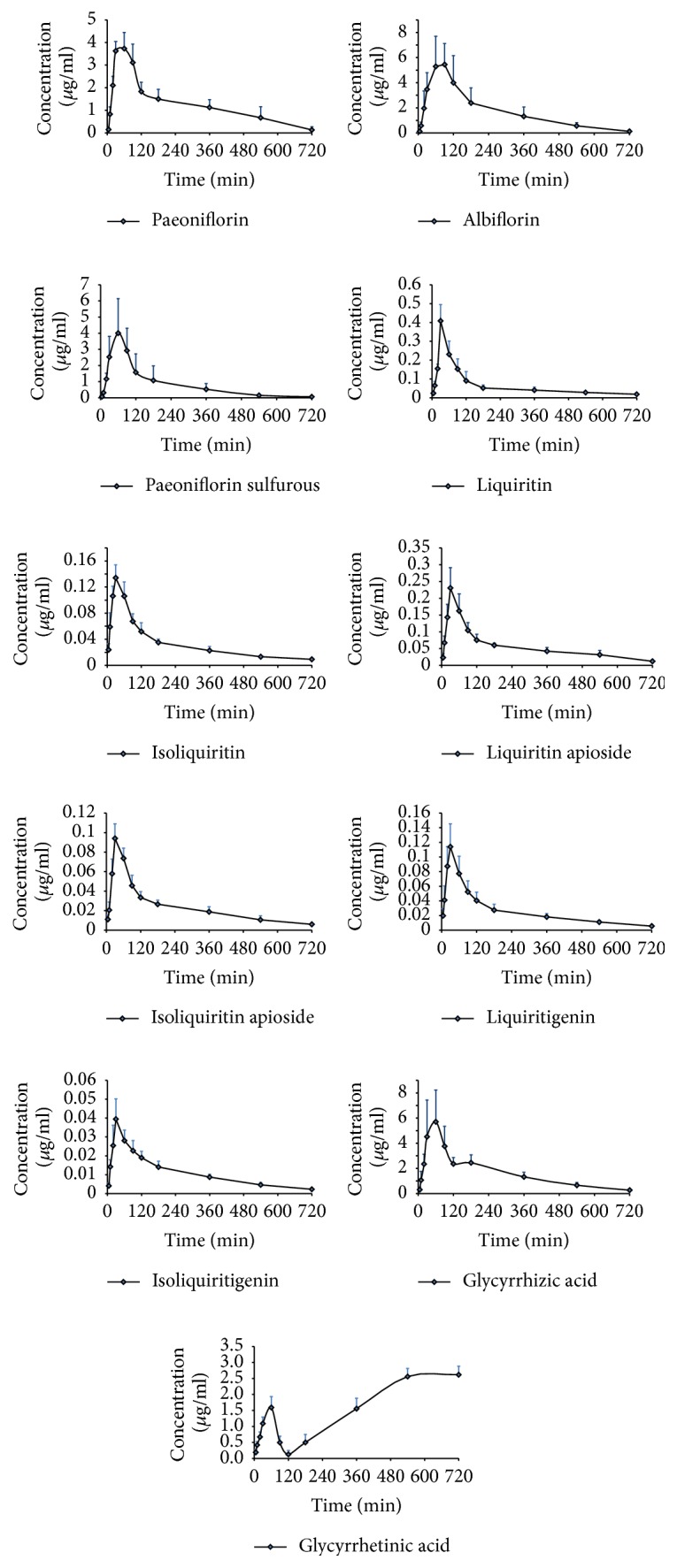
Concentration-time curve of 11 detected compounds in rats.

The validated UPLC-MS/MS method was successfully applied to the PK studies of eleven analytes following oral administration of GLGZG to individual male rats (*n* = 6). The main pharmacokinetic parameters were summarized in Table 8 and the mean plasma concentration-time profiles of the eleven analytes were illustrated in Figure 3.

As shown in Table 8 and Figure 3, the mean peak plasma time (*T*_max_) of liquiritin, isoliquiritin, liquirtin apioside, isoliquiritin apioside, liquiritigenin, and isoliquiritigenin was 26.67 min–35.00 min after oral administration which suggested moderately rapid absorption pattern. Their area under the concentration-time and bioactivity-time curves (AUC) was calculated and ranged from 8.52 mg·(L·min)^−1^ to 54.87 mg·(L·min)^−1^. Moreover, rate of elimination of liquiritin was slow with the half-life (*t*1/2) values 309.46 min. To our knowledge, the content of glycyrrhetinic acid was low but glycyrrhizic acid was high, and it was found that plasma concentration of glycyrrhetinic acid presented a trend of increasing; the phenomenon might be attributed to production of glycyrrhetinic acid in the process of glycyrrhizic acid metabolism in vivo. Existing literature suggests that glycyrrhizic acid can be hydrolyzed to obtain the glycyrrhetinic acid in vivo by removing two molecules of glucuronic acid [25]. In addition, there was a double-peak phenomenon of glycyrrhizic acid, which may be because of reabsorption or enterohepatic circulation. This phenomena match the experimental result of a pharmacokinetic study of licorice, which was executed by Qiao et al. [26]. *T*_max_ of liquiritin is consistent with the study by Kamei et al. researchers [27] and it was found that isoliquiritigenin was low in the blood. In addition, in comparison with the related literatures, *T*_max_ and *C*_max_ of liquirtin apioside, isoliquiritin apioside, and paeoniflorin sulfonate were extended or shortened in different degrees. However, further studies should be carried out to verify these phenomena and assumptions, and the drug metabolism evaluation and the detailed pharmacokinetic/pharmacodynamic studies of GLGZG need more studies. The information described this paper might be helpful for further studies of GLGZG and beneficial for application in clinical therapy.

## 4. Conclusion

The UPLC-MS/MS method developed and validated in this study was specific and sensitive, with acceptable accuracy and a short run time of 7 min for the simultaneous determination of liquiritin, isoliquiritin, liquirtin apioside, isoliquiritin apioside, liquiritigenin, isoliquiritigenin, glycyrrhizic acid, glycyrrhetinic acid, paeoniflorin, albiflorin, and paeoniflorin sulfonate together in vivo following the oral administration of GLGZG. The results might be helpful to provide certain references to clinical application of this medicine or others.

## Figures and Tables

**Table 1:** MS analysis of parameters of detected compounds and internal standards (ISs).

Analyte	RT (min)	[M − H]^−^ (*m/z*)	MS^*n*^ (*m/z*)
Paeoniflorin sulfonate (12)	2.18	543.11	121.02
Albiflorin (2)	3.67	525.16	121.02
Paeoniflorin (1)	3.90	525.16	121.02
Liquirtin apioside (5)	4.04	549.16	255.06
Liquiritin (3)	4.07	417.12	255.06
Isoliquiritin apioside (6)	4.26	549.16	255.06
Isoliquiritin (4)	4.33	417.12	255.06
Liquiritigenin (8)	4.56	255.06	135.01
Isoliquiritigenin (9)	4.91	255.06	119.05
Glycyrrhizic acid (10)	5.02	821.39	351.05
Glycyrrhetinic acid (11)	6.22	469.33	425.34
Ginsenoside Rb1 (IS1)	4.50	1107.59	1107.59
Swertiamarin (IS2)	2.77	419.11	179.05
Kaempferol-3-O-rutinoside (IS3)	4.16	593.15	285.04

**Table 2:** Calibration curves and linear ranges of detected compounds (*n* = 8).

Analyte	Regression equation	*r*	Linear range (*μ*g/mL)
Paeoniflorin	*y* = 0.381*x* − 0.009	*r* = 0.9980	0.040–8.00
Albiflorin	*y* = 1.341*x* − 0.052	*r* = 0.9597	0.040–10.00
Liquiritin	*y* = 112.3*x* − 1.519	*r* = 0.9930	0.005–1.00
Isoliquiritin	*y* = 6.612*x* − 0.065	*r* = 0.9644	0.005–0.50
Liquirtin apioside	*y* = 36.36*x* − 0.222	*r* = 0.9970	0.005–0.50
Isoliquiritin apioside	*y* = 6.044*x* − 0.072	*r* = 0.9737	0.005–0.50
Liquiritigenin	*y* = 123.0*x* − 1.408	*r* = 0.9910	0.001–0.20
Isoliquiritigenin	*y* = 52.37*x* − 0.980	*r* = 0.9813	0.001–0.20
Glycyrrhizic acid	*y* = 12.12*x* − 0.687	*r* = 0.9813	0.050–1.00
Glycyrrhetinic acid	*y* = 2.443*x* + 0.019	*r* = 0.9985	0.010–4.00
Paeoniflorin sulfonate	*y* = 7.091*x* − 0.112	*r* = 0.9985	0.050–11.25

**Table 3:** LLOQ and LLOD of detected compounds.

Analyte	Linear range (*μ*g·mL^−1^)	LLOQ (ng·mL^−1^)	LLOD (ng·mL^−1^)
Paeoniflorin	0.04–8	11.10	3.32
Albiflorin	0.04–10	5.42	1.73
Liquiritin	0.005–1	0.11	0.03
Isoliquiritin	0.005–0.5	0.81	0.26
Liquirtin apioside	0.005–0.5	0.46	0.14
Isoliquiritin apioside	0.005–0.5	0.88	0.36
Liquiritigenin	0.001–0.2	0.55	0.20
Isoliquiritigenin	0.001–0.2	0.93	0.36
Glycyrrhizic acid	0.05–10	29.82	10.67
Glycyrrhetinic acid	0.01–4	15.74	5.95
Paeoniflorin sulfonate	0.05–11.25	4.21	1.52

**Table 4:** The precision and accuracy of detected compounds in rat plasma (*n* = 6).

Analyte	Nominal concentration (*μ*g·mL^−1^)	Intraday precision /RSD	Interday precision /RSD	Intraday accuracy	Interday accuracy
Paeoniflorin	0.1	4.98%	5.52%	97.35%	96.93%
	2	4.44%	3.33%	92.48%	92.08%
	8	3.77%	3.46%	107.09%	106.62%
Albiflorin	0.1	4.28%	4.01%	103.05%	101.85%
	2	2.87%	4.75%	108.45%	101.05%
	8	3.47%	3.76%	113.36%	96.00%
Liquiritin	0.01	5.53%	4.68%	99.15%	104.48%
	0.2	5.28%	3.62%	94.19%	109.29%
	0.5	3.32%	4.31%	97.90%	105.66%
Isoliquiritin	0.01	6.12%	6.18%	113.25%	96.76%
	0.1	3.44%	3.47%	109.07%	93.48%
	0.25	3.62%	4.56%	99.60%	108.24%
Liquirtin apioside	0.01	4.09%	4.68%	99.95%	94.98%
	0.1	5.59%	4.44%	114.03%	90.23%
	0.25	4.38%	4.42%	109.95%	98.40%
Isoliquiritin apioside	0.01	5.87%	1.67%	103.66%	100.55%
	0.1	2.58%	3.15%	98.48%	95.52%
	0.25	4.07%	3.87%	98.95%	95.14%
Liquiritigenin	0.005	4.62%	4.01%	94.95%	96.74%
	0.1	4.87%	5.13%	94.62%	94.19%
	0.2	2.57%	5.14%	109.56%	106.41%
Isoliquiritigenin	0.005	6.41%	5.85%	102.95%	112.04%
	0.02	4.95%	4.87%	97.80%	110.61%
	0.05	5.13%	4.30%	94.00%	91.25%
Glycyrrhizic acid	0.1	2.21%	2.84%	102.05%	99.15%
	2	4.18%	3.16%	96.95%	91.90%
	8	4.03%	4.32%	112.26%	109.07%
Glycyrrhetinic acid	0.01	3.58%	4.01%	98.59%	100.15%
	1	5.88%	4.31%	108.85%	111.16%
	2	2.97%	5.19%	92.82%	110.17%
Paeoniflorin sulfonate	0.1	3.90%	5.85%	97.71%	99.35%
	2	3.54%	4.72%	93.66%	94.38%
	8	3.62%	5.15%	107.48%	96.05%

**Table 5:** The stability of detected compounds in rat plasma (*n* = 6).

Analyte	Nominal concentration (*μ*g·mL^−1^)	Autosampler stability (24 h)		Stability (24 h at room temperature)		Freeze-thaw stability (3 cycles)	
		Mean	RSD	Mean	RSD	Mean	RSD
Paeoniflorin	0.1	103.46%	5.06%	105.66%	2.67%	97.71%	2.01%
	2	98.29%	5.07%	91.25%	4.29%	92.82%	4.30%
	8	113.81%	3.60%	109.75%	3.44%	107.48%	3.77%

Albiflorin	0.1	97.93%	3.01%	94.78%	1.84%	99.77%	4.44%
	2	111.49%	4.73%	93.66%	5.36%	108.14%	4.44%
	8	107.72%	2.72%	108.45%	4.47%	98.89%	4.68%

Liquiritin	0.01	112.48%	3.87%	95.70%	4.18%	101.75%	5.02%
	0.2	92.30%	3.83%	110.81%	3.68%	109.75%	4.11%
	0.5	90.84%	5.13%	106.73%	2.84%	97.91%	3.02%

Isoliquiritin	0.01	105.18%	3.83%	100.74%	3.18%	98.98%	4.61%
	0.1	97.14%	3.44%	99.28%	5.11%	94.03%	4.08%
	0.25	95.62%	4.53%	98.59%	3.58%	108.88%	2.72%

Liquirtin apioside	0.01	95.66%	4.46%	111.24%	3.77%	107.20%	3.24%
	0.1	90.88%	3.99%	96.07%	4.81%	93.95%	2.87%
	0.25	105.23%	4.53%	104.43%	4.13%	94.78%	4.68%

Isoliquiritin apioside	0.01	102.25%	5.16%	101.13%	3.82%	109.53%	4.27%
	0.1	106.88%	3.81%	101.45%	3.31%	93.01%	4.87%
	0.25	112.48%	4.68%	98.59%	3.76%	96.66%	2.11%

Liquiritigenin	0.005	98.45%	3.58%	111.60%	4.41%	97.45%	6.59%
	0.1	93.53%	5.60%	108.45%	4.01%	93.29%	5.43%
	0.2	97.16%	3.17%	97.03%	3.73%	108.02%	4.68%

Isoliquiritigenin	0.005	106.19%	5.84%	99.77%	5.88%	99.28%	3.01%
	0.02	91.71%	2.78%	93.66%	2.58%	107.70%	2.82%
	0.05	108.30%	4.07%	100.94%	2.85%	109.21%	5.13%

Glycyrrhizic acid	0.1	97.71%	3.80%	96.05%	5.57%	98.31%	2.87%
	2	92.82%	5.28%	92.18%	3.68%	108.78%	4.87%
	8	107.48%	4.98%	94.94%	5.41%	98.20%	4.07%

Glycyrrhetinic acid	0.01	102.25%	3.35%	111.03%	4.54%	111.93%	4.95%
	1	97.14%	3.34%	95.89%	3.72%	92.58%	2.44%
	2	96.54%	5.64%	90.19%	4.81%	94.32%	3.02%

Paeoniflorin sulfonate	0.1	102.25%	4.44%	109.21%	2.23%	99.57%	6.05%
	2	96.28%	4.30%	94.32%	5.67%	94.59%	4.82%
	8	93.03%	5.43%	96.38%	2.25%	93.39%	5.13%

**Table 6:** The extraction recovery of detected compounds in rat plasma (*n* = 6).

Analyte	Nominal concentration (*μ*g·mL^−1^)	Extraction recovery		
		Mean	SD	RSD
Paeoniflorin	0.1	95.62%	9.60%	10.04%
	2	89.08%	8.29%	9.31%
	8	88.65%	5.87%	6.62%
Albiflorin	0.1	95.43%	8.97%	9.40%
	2	95.33%	10.93%	11.47%
	8	92.33%	8.32%	9.01%
Liquiritin	0.01	104.13%	11.50%	11.05%
	0.2	85.14%	7.70%	9.04%
	0.5	96.98%	9.43%	9.72%
Isoliquiritin	0.01	90.56%	7.54%	8.32%
	0.1	86.10%	8.39%	9.74%
	0.25	91.48%	4.68%	5.12%
Liquirtin apioside	0.01	90.38%	8.90%	9.85%
	0.1	99.47%	6.93%	6.96%
	0.25	90.76%	10.23%	11.27%
Isoliquiritin apioside	0.01	81.30%	7.70%	9.47%
	0.1	108.22%	7.52%	6.95%
	0.25	93.61%	9.43%	10.07%
Liquiritigenin	0.005	94.50%	8.51%	9.01%
	0.1	88.01%	6.42%	7.29%
	0.2	92.48%	6.32%	6.83%
Isoliquiritigenin	0.005	102.31%	9.49%	9.27%
	0.02	101.35%	9.76%	9.63%
	0.05	90.08%	5.19%	5.76%
Glycyrrhizic acid	0.1	93.11%	8.21%	8.82%
	2	100.35%	9.58%	9.54%
	8	99.46%	6.38%	6.41%
Glycyrrhetinic acid	0.01	106.28%	7.13%	6.71%
	1	87.71%	7.04%	8.02%
	2	103.53%	8.93%	8.63%
Paeoniflorin sulfonate	0.1	95.33%	7.32%	7.68%
	2	98.05%	6.42%	6.55%
	8	104.21%	8.45%	8.11%

**Table 7:** The matrix effect of detected compounds in rat plasma.

Analyte	Nominal concentration (*μ*g·mL^−1^)	Matrix effect		
		Mean	SD	RSD
Paeoniflorin	0.1	104.05%	4.16%	4.00%
	2	93.79%	5.89%	6.28%
	8	98.33%	6.01%	6.11%
Albiflorin	0.1	101.59%	4.26%	4.19%
	2	107.86%	9.43%	8.74%
	8	99.29%	5.97%	6.02%
Liquiritin	0.01	88.91%	6.93%	7.79%
	0.2	106.80%	7.42%	6.95%
	0.5	102.29%	5.04%	4.92%
Isoliquiritin	0.01	97.40%	5.23%	5.37%
	0.1	91.78%	4.15%	4.52%
	0.25	98.38%	9.32%	9.48%
Liquirtin apioside	0.01	113.32%	5.30%	4.68%
	0.1	105.87%	6.20%	5.86%
	0.25	99.60%	5.64%	5.66%
Isoliquiritin apioside	0.01	110.04%	3.08%	2.80%
	0.1	106.10%	3.46%	3.27%
	0.25	100.67%	4.21%	4.18%
Liquiritigenin	0.005	89.86%	6.43%	7.16%
	0.1	94.92%	6.30%	6.64%
	0.2	99.45%	6.61%	6.65%
Isoliquiritigenin	0.005	108.12%	7.51%	6.94%
	0.02	101.51%	8.30%	8.18%
	0.05	96.87%	4.63%	4.78%
Glycyrrhizic acid	0.1	99.71%	8.42%	8.44%
	2	90.76%	8.72%	9.61%
	8	106.96%	8.42%	7.87%
Glycyrrhetinic acid	0.01	95.51%	6.25%	6.55%
	1	93.47%	4.64%	4.97%
	2	101.34%	5.93%	5.85%
Paeoniflorin sulfonate	0.1	100.75%	8.54%	8.48%
	2	104.38%	7.23%	6.93%
	8	110.07%	6.31%	5.73%

**Table 8:** Pharmacokinetic parameters of 11 compounds in rat plasma in Gualou Guizhi decoction (*n* = 6).

Analyte	*C*_max_ (mg·mL^−1^)	*T*_max_ (min)	*T*_1/2*z*_ (min)	AUC_(0–*∞*)_ (mg·(L·min)^−1^)	MRT_(0–*∞*)_ (min)	*Vz*/*F* (L·kg^−1^)
Paeoniflorin	4.16 ± 0.72	80.00 ± 17.32	142.98 ± 30.11	904.73 ± 317.76	235.91 ± 40.45	6.31 ± 1.63
Albiflorin	6.41 ± 2.01	60.00 ± 21.21	127.68 ± 35.74	1263.37 ± 306.91	215.15 ± 45.14	2.67 ± 0.87
Liquiritin	0.41 ± 0.09	30.00 ± 0.00	309.46 ± 66.39	54.87 ± 14.40	355.95 ± 72.55	167.40 ± 35.86
Isoliquiritin	0.15 ± 0.04	28.33 ± 4.08	276.19 ± 51.94	27.17 ± 2.69	339.62 ± 46.83	297.43 ± 70.05
Liquirtin apioside	0.23 ± 0.06	30.00 ± 0.00	234.63 ± 60.70	44.70 ± 9.12	319.49 ± 61.75	152.93 ± 31.84
Isoliquiritin apioside	0.11 ± 0.02	35.00 ± 12.25	244.62 ± 34.16	18.90 ± 3.20	315.50 ± 43.96	378.57 ± 56.73
Liquiritigenin	0.12 ± 0.03	26.67 ± 5.16	232.26 ± 28.65	20.38 ± 4.30	295.11 ± 25.08	340.80 ± 73.56
Isoliquiritigenin	0.04 ± 0.01	30.00 ± 0.00	203.86 ± 40.50	8.52 ± 0.98	285.44 ± 50.94	700.08 ± 187.21
Glycyrrhizic acid	4.37 ± 1.49	54.00 ± 13.42	171.54 ± 33.67	1262.61 ± 158.73	264.02 ± 49.72	3.92 ± 1.44
Glycyrrhetinic acid	2.92 ± 0.67	600 ± 146.97	132.11 ± 28.33	2041.10 ± 706.58	709.84 ± 83.32	2.91 ± 1.44
Paeoniflorin sulfonate	4.50 ± 1.06	80.00 ± 30.11	142.98 ± 30.11	777.42 ± 163.06	235.91 ± 40.45	6.31 ± 1.63
